# P-2002. Proportions and Taxonomic Diversity of Non-Aspergillus Molds Detected Using Plasma Microbial Cell-free DNA Sequencing from 2018–2024

**DOI:** 10.1093/ofid/ofaf695.2166

**Published:** 2026-01-11

**Authors:** Paul Lewis, Monica Shah, David Berman, Sarah Y Park, Eliza J Chang, Claire Dysart

**Affiliations:** Karius, Redwood City, CA; Karius, Redwood City, CA; Karius, Redwood City, CA; Karius, Inc., Redwood City, California; Karius, Redwood City, CA; Karius, Redwood City, CA

## Abstract

**Background:**

Diagnosing non-Aspergillus invasive mold infections (NAIMIs) can be challenging given nonspecific clinical findings and radiological signs, low culture yields, and lack of commercial nonculture-based tests. In 2025, the American Society of Transplantation and Cellular Therapy (ASTCT) published practice guidelines for managing and preventing NAIMIs in hematopoietic cell transplant recipients and recognized considering microbial cell-free DNA (mcfDNA) sequencing “in selected cases where diagnosis is particularly challenging.” We reviewed NAIMI pathogen detections using Karius Spectrum™, a validated, real-time, pathogen-agnostic mcfDNA sequencing test used in hospital settings.

**Methods:**

We reviewed and described NAIMI pathogens detected and quantified in all US patients’ plasma samples, submitted to the Karius CLIA certified/CAP accredited laboratory from Apr 2018–Nov 2024. Results were categorized into 4 groups based on ASTCT specifications: 1) Mucorales; 2) Fusarium spp; 3) Scedosporium / Lomentospora; and 4) rare molds.

**Results:**

Of 78,143 patients tested during the timeframe, 1,146 (1.5%) patients had 1,289 positive results for a NAIMI pathogen. Mucorales occurred most frequently (904, 70.1%), followed by rare molds (174, 13.5%), Fusarium spp (131, 10.2%), and Scedosporium / Lomentospora (80, 6.2%). 16 different genera were detected, the most common being Rhizopus (n=485), Rhizomucor (n=195), and Fusarium (n=131), and 45 unique taxa were identified (most common species: Rhizopus microsporus, n=224; Rhizopus arrhizus/oryzae, n=175; and Rhizomucor pusillus, n=155).

**Conclusion:**

Plasma mcfDNA sequencing provides a pathogen-agnostic approach for NAIMIs that are otherwise difficult to diagnose. Further studies are needed to assess the patient distribution, characteristics, and underlying conditions as well as the clinical impact of NAIMI mcfDNA detections on patient care.

**Disclosures:**

Paul Lewis, PharmD, Karius: Karius employee Monica Shah, PharmD, Karius: Karius employee David Berman, DO, Karius: Karius employee Sarah Y. Park, MD, FAAP, Karius, Inc.: current employee Eliza J. Chang, A.B., Karius, Inc.: current employee Claire Dysart, PharmD, Karius: Karius employee

